# In Silico Drug Prescription for Targeting Cancer Patient Heterogeneity and Prediction of Clinical Outcome

**DOI:** 10.3390/cancers11091361

**Published:** 2019-09-13

**Authors:** Elena Piñeiro-Yáñez, María José Jiménez-Santos, Gonzalo Gómez-López, Fátima Al-Shahrour

**Affiliations:** Bioinformatics Unit, Spanish National Cancer Research Centre (CNIO), 28029 Madrid, Spain; epineiro@cnio.es (E.P.-Y.); mjjimenez@cnio.es (M.J.J.-S.)

**Keywords:** precision medicine, cancer genomics, intra-tumour heterogeneity, in silico prescription, bioinformatics, pharmacogenomics, druggable genome

## Abstract

In silico drug prescription tools for precision cancer medicine can match molecular alterations with tailored candidate treatments. These methodologies require large and well-annotated datasets to systematically evaluate their performance, but this is currently constrained by the lack of complete patient clinicopathological data. Moreover, in silico drug prescription performance could be improved by integrating additional tumour information layers like intra-tumour heterogeneity (ITH) which has been related to drug response and tumour progression. PanDrugs is an in silico drug prescription method which prioritizes anticancer drugs combining both biological and clinical evidence. We have systematically evaluated PanDrugs in the Genomic Data Commons repository (GDC). Our results showed that PanDrugs is able to establish an a priori stratification of cancer patients treated with Epidermal Growth Factor Receptor (EGFR) inhibitors. Patients labelled as responders according to PanDrugs predictions showed a significantly increased overall survival (OS) compared to non-responders. PanDrugs was also able to suggest alternative tailored treatments for non-responder patients. Additionally, PanDrugs usefulness was assessed considering spatial and temporal ITH in cancer patients and showed that ITH can be approached therapeutically proposing drugs or combinations potentially capable of targeting the clonal diversity. In summary, this study is a proof of concept where PanDrugs predictions have been correlated to OS and can be useful to manage ITH in patients while increasing therapeutic options and demonstrating its clinical utility.

## 1. Introduction

Large-scale cancer genome projects such as The Cancer Genome Atlas (TCGA) and The International Cancer Genome Consortium (ICGC) have revealed that cancers are characterized by a high multidimensional genomic heterogeneity among different tumours and also within the same patient [1]. Tumour genomics heterogeneity explains that the majority of cancers are not single diseases but rather an array of disorders with distinct molecular mechanisms [2].

It is widely admitted that the massive analysis and integration of patients’ genomics profiles and clinical data will promote cancer precision medicine approaches [3,4] by guiding the development of prognostic, diagnostic and therapeutic strategies in cancer. Significant progress towards this goal has been made by exploiting the data collection and analysis efforts of large cancer genomics consortia such as TCGA and ICGC. Unfortunately, these discoveries are limited by the lack of patient clinical information and the need of novel methodologies to successfully achieve the precision medicine challenges.

Currently, there is a large catalogue of bioinformatics methods to evaluate and interpret cancer genomic landscapes. Amongst such methods, in silico drug prescription tools have recently emerged to prioritize patients’ specific genomic alterations with matched therapies and candidate drugs [5,6,7,8]. However, the systematic evaluation of their performance is currently constrained by the lack of standardised information and accessible clinical data of the patients enrolled in cancer genome consortia (e.g., the treatments received, measure of response, survival, etc.). In response to these challenges, the National Cancer Institute (NCI) Genomic Data Commons effort (GDC) has recently provided a valuable repository containing unified and standardised clinic-genomic data for patients included in the NCI cancer research programs [9]. GDC provides data access and enables data sharing from diverse types of genomics studies such as TCGA [10], Therapeutically Applicable Research to Generate Effective Treatments (TARGET) [11] and Cancer Cell Line Encyclopaedia (CCLE) [12] and their associated phenotypic/clinical information. Other genomics/personalized medicine initiatives have been launched around the world [13,14,15]. An interesting multicentre project is ICGC-ARGO (Accelerate Research in Genomic Oncology) which aims to collect a much richer dataset of cancer genomes with clinical information, health and response to therapy.

Another current challenge in translational cancer genomics is tackling intra-tumour heterogeneity (ITH) and cancer evolution since ITH has recently been revealed as a key factor in cancer patients’ outcome contributing to the failure in the use of therapies [16,17], the appearance of drug resistance [18,19], leading to different responses [20,21] and, therefore, a higher lethality rate. ITH arises as a result of the evolving process tumoural cells suffer during their growth and propagation. ITH can be spatial (variability in different locations) and temporal (variability over the temporal evolution of the tumour) [22]. Nevertheless, current common clinical practice does not take into account ITH, using exclusively a therapy administration strategy based on bulk analysis results and limiting the effectiveness of the treatments. What can happen in these cases is that the tumoural cells underlying sequenced clones could be favoured by the administration of the treatment and settle, perpetuating the tumour [23,24]. Incorporating ITH assessment to other molecular profiling approaches would add more resolution on the study of tumour biology and evolution. Moreover, understanding ITH would be valuable for designing successful therapeutic strategies in the context of precision oncology paradigm [25,26]. For this purpose, TRAcking Cancer Evolution through therapy (Rx) initiative (TRACERx) has been recently launched to relate ITH with clinical outcome. It is expected that TRACERx findings will provide a comprehensive information of ITH impact in patients from diagnosis through to relapse in the near future.

Bioinformatics approaches have been recently developed to dissect ITH and its consequences on cancer evolution [27,28,29,30]. Only a few tools to predict combination therapy using ITH have been developed [31,32]. Moreover, systematic computational efforts to consider ITH on choosing therapies are extremely rare [18,33], besides these studies do not directly assess the impact of ITH on their treatment predictions.

In this work, we will evaluate the results generated by PanDrugs [7], a bioinformatics platform developed in our laboratory to prioritize anticancer drug treatments according to individual genomic data employing GDC standardised data. This will allow us to relate PanDrugs predictions to patients’ genomic variability, drug response and survival outcome. Additionally, we will use PanDrugs to design anticancer treatment regimens considering temporal and spatial ITH using public sequencing data obtained from acute myeloid leukaemia (AML) and non-small cell lung cancer (NSCLC) cancer patients [34,35].

## 2. Results

We tested PanDrugs applicability in sequencing data to relate the proposed therapeutic options with survival outcome and evaluate its impact considering ITH.

PanDrugs is an in silico drug prescription method that identifies druggable genomic alterations and prioritizes drug therapies based on clinical, biological and pharmacological evidence [7]. To do so, PanDrugs mines PanDrugsdb, a database with information about the implication of genes in different types of cancer and the effectiveness of drugs that target those mutated genes. The current version of PanDrugsdb stores 56,297 drug-target associations obtained from 4804 genes and 9092 drugs. When the user inputs the type tumour and a list of mutated genes or a Variant Calling Format (VCF) file with somatic variants, PanDrugs calculates two scores integrating clinical, biological and pharmacological sources and databases to propose tailored anticancer therapies based on somatic alterations: i) Gene Score (GScore) ranging between 0 and 1 based on the evidence supporting gene clinical implication and its biological relevance in cancer and ii) Drug Score (DScore) ranges between −1 and 1 and estimates drug response (resistance: negative values; sensitivity: positive values) and treatment suitability. Besides PanDrugs classifies druggable genes as (a) direct targets, genes that can be directly targeted by a drug, (b) biomarkers, genes which genetic status is associated with a drug response and (c) pathway member, a targetable gene located downstream to the altered one. PanDrugs output provides a prioritized list of candidate drugs considering GScore and DScore values that will support their effectiveness in cancer treatment. Those drugs with higher DScores targeting genes with GScores closer to 1 are suggested as the Best Therapeutic Candidates for that particular patient. Thus, PanDrugs offers a valuable in silico drug prescription tool that helps the genomics profile interpretation and it might improve clinical decision making.

### 2.1. A priori Cancer Patients’ Stratification Using PanDrugs

**Figure 1:**
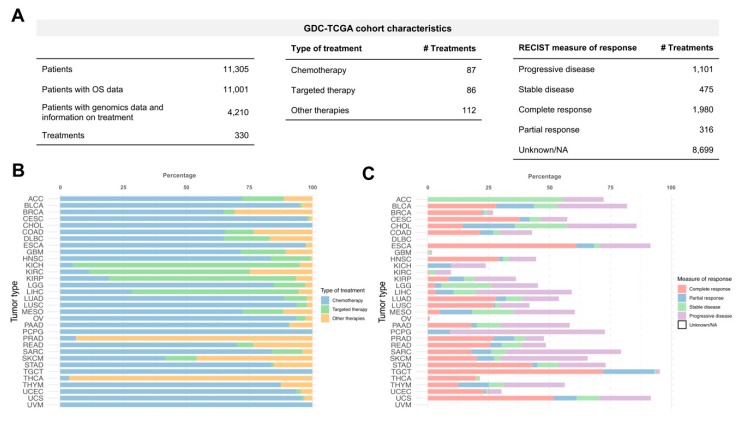
Genomic Data Commons effort and The Cancer Genome Atlas (GDC-TCGA) cohort description. (**A**) Tables showing total number of patients and drugs administered, type of treatments received and Response Evaluation Criteria in Solid Tumours (RECIST) measure of response in GDC-TCGA cohort. (**B**) Percentage of treatments received by tumour type. Bar colour represents the type of anticancer therapy (targeted therapy, chemotherapy and other therapies such as hormone therapy, immunotherapy and ancillary therapies). (**C**) Percentage of treatments with measure of response by tumour type. Bar colour represents the different measures of response following the RECIST guidelines.

We used the TCGA cohort extracted from the GDC Portal to predict cancer patients drug response considering inter-tumoral heterogeneity. At the time of the analysis, the GDC-TCGA cohort contained 11,305 patients over 33 different tumour types. All the sequenced samples were untreated primary tumors. Most of the cases (11,001) have overall survival (OS) data as clinical outcome endpoint. 4210 patients (37.2%) have available genomics data (mutations and copy number variation) and information on treatment received (Figure 1A). A total of 330 different treatments were administered and belong to several types of cancer therapy including targeted therapy (26.1%), chemotherapy (26.4%) and other therapies (33.9%) like immunotherapy or hormone therapy (Figure 1A). Most of the patients (83.7%) received chemotherapy during their treatment regimens (first and second-line treatments, etc). Although, there are some tumour types where the treatments were mostly based on targeted therapies such as renal cancers (Kidney Chromophobe, KICH; Kidney renal clear cell carcinoma, KIRC; Kidney renal papillary cell carcinoma, KIRP) and Liver Hepatocellular Carcinoma (LIHC). Other types of treatment such as hormone therapy were the predominant therapies for prostate adenocarcinoma (PRAD) and thyroid carcinoma (THCA) (Figure 1B). Interestingly, GDC provides the measure of patient response for each therapy and their time to relapse (Figure 1A) that could be used to relate drug responses with survival outcome. However, the measure of response classified by progressive disease, stable disease, complete response or partial response is available only for 40.4% of the cases (Figure 1C). It is important to mention that there are tumour types that do not have data related to clinical response (i.e., Lymphoid Neoplasm Diffuse Large B-cell Lymphoma (DLBC) and Uveal Melanoma (UVM)) which reflects the lack of standardization and the difficulty of collecting and managing clinical information in large-scale studies.

**Figure 2:**
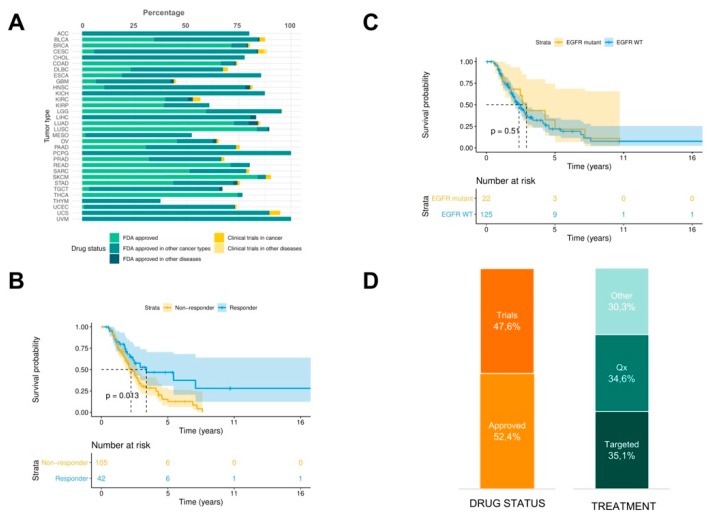
PanDrugs analysis in the Genomic Data Commons effort and The Cancer Genome Atlas (GDC-TCGA) cohort. (**A**) PanDrugs in silico prescription results for each tumour type in GDC-TCGA cohort. Bar colour represents drug status. Only those treatments showing Gene Score > 0.6 were considered. (**B**) Kaplan–Meier plot for responders and non-responders to EGFR inhibitor (EGFRi) treatment. Responders include cases identified by PanDrugs as a priori sensitive to the treatment. Non-responders include cases identified by PanDrugs as a priori resistant or without evidence of drug response. (**C**) Kaplan–Meier plot for EGFR-mutant and EGFR wild type cases treated with EGFR inhibitors. (**D**) PanDrugs prescription for EGFRi non-responder patients by drug status and type of treatment. (Qx: chemotherapy).

In order to propose in silico drug prescriptions, PanDrugs was systematically applied on the GDC-TCGA cohort of 4210 patients whose genomic data (mutations and copy number variation, CNV) and survival outcome was available. PanDrugs results showed that an in silico prescription of Food and Drug Administration (FDA) approved drugs offered treatments for 73.8% of patients when point mutations, indels and CNVs were considered simultaneously and 1.2% of patients were prescribed with drugs in current clinical trials (Figure 2A).

Then, we were interested in evaluating whether PanDrugs recommendations could drive a priori patient’s stratification by responders or non-responders to a specific drug and relate it with their overall survival (OS). To illustrate this, we focused on those patients (*n* = 147) who received during their treatment regimens of EGFR inhibitors (EGFRi) including monoclonal antibodies (i.e., cetuximab, panitumumab, etc.) or small molecule tyrosine kinase inhibitors (i.e., erlotinib, gefitinib, etc). We selected EGFRi because they represent targeted therapies with regulatory approval for mutant-driven tumours and adopted in the clinical guidelines. Using the molecular profiles (mutations and CNV) of these patients, PanDrugs was executed to identify which genomic alterations are associated with sensitivity to EGFRi.

Based on PanDrugs results, patients were categorised into three types according to their known molecular evidence associated to drug response: a) patients harbouring drug sensitivity mutations and no drug resistance mutations associated to EGFRi response, b) patients harbouring drug resistance mutations associated to EGFRi response and c) patients without molecular evidence of drug response neither sensitive nor resistant to EGFRi. The first group of patients was labelled as “responders” and the other two groups as “non-responders”.

Next to PanDrugs a priori patients’ stratification, we performed OS analysis between responder and non-responder groups. The resulting Kaplan–Meier plot shows that the patients classified as responders have a significantly increased OS (*p*-value = 0.013) compared to non-responders (Figure 2B). This suggests that non-responders could be considered as patients with pre-existing pharmacological resistance based on their molecular evidence (i.e., drug resistance mutations) and their worse prognosis could suggest an earlier relapse.

Next, we wanted to address if this result is biased by EGFR mutational status where EGFRis are expected to show more efficacy in EGFR-mutant than EGFR wild-type cases a priori. Figure 2C shows that OS outcome is not dependent on EGFR mutational status (*p* = 0.51). This result highlights the importance of taking into account the whole molecular profile instead of using an individual biomarker to guide the choice of the therapy to be administered. Based on previous studies [36], it is expected that around 70% of EGFR mutant patients will show a partial response to EGFRi following RECIST guidelines and the remaining 30% will not respond to the treatment. PanDrugs is able to stratify patients a priori with distinct prognosis based on the detection of drug resistant and sensitivity associated mutations. Notably, PanDrugs was able to propose alternative therapies for 48.5% patients who were initially classified by their molecular profile as non-responder (45.2% were FDA approved drugs and 54.8% in clinical trials). Amongst them, 43.1% were targeted therapies and 36.2% chemotherapies (Figure 2D). This result could open a window of therapeutic opportunities for *a priori* classified non-responder patients.

### 2.2. In Silico Drug Prescription Considering Intra-tumour Heterogeneity (ITH)

We studied ITH considering temporal and spatial evolution to evaluate the tumour therapeutic complexity compared to clonal and sub-clonal heterogeneity and the scope of tumour cells whose therapeutic approach could be targeted with approved drugs, clinical trials or drug repositioning strategies using PanDrugs.

#### 2.2.1. PanDrugs Prescription in Temporal ITH on AML Patient Genomes

At the time of tumour diagnosis, pre-existing drug resistance clones are commonly found at low variant allele frequencies (VAF) and can be maintained or expanded during treatment [37]. These pre-existing clones can be related to patient drug responses, intrinsic drug resistance and their identification may indicate promising drug targets. Considering temporal clonal evolution, we could propose a rationale for drug administration (i.e., first and second-line treatment).

To assess the therapeutic impact of the temporal clonal evolution we used the whole genome sequencing data of the primary and relapse tumour of an acute myeloid leukaemia (AML) patient (patient ID: UPN933124) obtained from the Ding et al. publication [34]. Briefly, AML is a cancer of the myeloid line of blood cells, initiated and driven by mutations in the genome. The standard treatment for AML is chemotherapy: induction therapy to achieve remission, followed by consolidation therapy to eliminate any residual disease. Despite these multiple therapies, AML patients often relapse. For most types of AML, the remission rate is around 67% and those older than 60 do not typically respond to treatment with a 27.4% overall five-year survival rate. Thus, there is an urgent need to identify tumour-specific molecular alterations that could lead to the development of new targeted therapies to help in clinical decision making.

In Ding et al.’s study, the authors sequenced the complete genomes of primary tumours, relapsed tumours, and matched normal (skin) samples from eight AML patients to study clonal evolution at the genetic level. One of these eight cases was from patient UPN933124, whose primary tumour was the first cancer genome to be published [38]. Bulk whole-genome sequencing results of primary and relapsed tumours showed that there were 413 validated somatic events in UPN933124, of which 78 were relapse-specific, five were primary-tumour-specific, and 330 were shared between tumours. Interestingly, the authors concluded that most of the somatic events found in the primary tumour were also present in the relapse and vice versa. Authors presented a tumour evolution model in UPN933124 which was based on mutant allele frequencies and suggested that there were several tumour subpopulations in the primary tumour defined by distinct sets of mutations. This model suggested that a relatively minor subpopulation of tumour cells survived chemotherapy and arose to become the dominant subclone at relapse. In the process, it gained additional mutations, possibly via the DNA damage induced by chemotherapy. Thus, it would be interesting to know beforehand the presence of these pre-existing drug resistant clones and along with methods to identify mutated drug targets will allow us to suggest drugs that could have been administered to the patient after chemotherapy or in combination to delay or prevent relapse.

**Figure 3:**
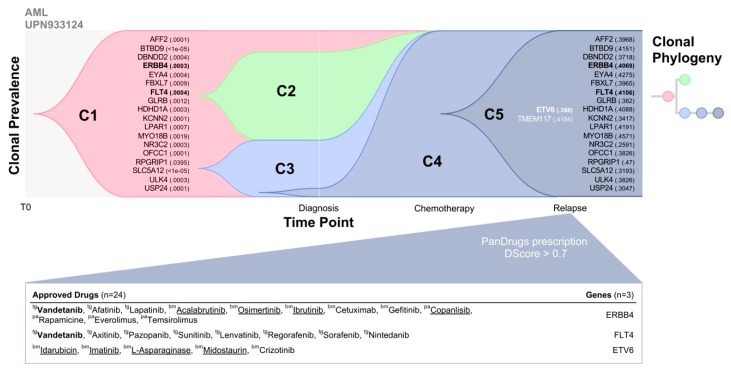
Intra-tumour heterogeneity (ITH) temporal analysis and PanDrugs prescription. TimeScape representation shows the clonal evolution for acute myeloid leukaemia (AML) patient UPN933124. Clonal prevalence (vertical axis) are plotted across timepoints for each clone (C1–C5). The evolutionary relationships between clones are captured by the phylogenetic tree. Variant allele frequency (VAF) is reported in parentheses and those genes that were clone-specific are written in white. Three genes (*ERBB4*, *FLT4* and *ETV6*), highlighted in bold, had a sensitivity response to Food and Drug Administration (FDA) approved drugs with a Drug Score > 0.7, which are detailed in the table below. Those drugs directed against several targets are written in bold and those approved for blood cancer are underlined. The type of target for each drug is reported (tg: direct target; bm; biomarker; pa: pathway member).

To evaluate potential therapeutic options in UPN933124, we applied PanDrugs using the pre-existing mutations detected in a primary tumour at low VAF (<1%) which are also present at higher VAF (30–40%) in relapse. First, we used TimeScape, a tool for navigating clonal dynamics over time [39] that integrates UPN933124 somatic mutations and their allele prevalence observed in each clone previously reported in Ding et al. Clonal prevalence at two time points, including the diagnosis and relapse after chemotherapy administration shows five different clones (Figure 3). Clone 5 (C5) represents the minority drug resistant clone at diagnosis that was positively selected after chemotherapy administration increasing their frequency and becoming the dominant clone that caused disease relapse. Eighteen mutated genes were positively selected in the relapse tumour which were already present in the primary tumour (diagnosis) at low VAF (<1%) and 2 genes were relapse-specific (*ETV6* and *TMEM117*). Then, PanDrugs was executed using as input these 20 mutated genes where 10 of such genes were druggable (Tables S1 and S2). Interestingly, PanDrugs provided in silico drug prescription (DScore > 0.7) for known targetable genes such as *ERBB4*, *FLT4* and *ETV6* (Figure 3). Drugs approved for AML such as acalabrutinib, osimertinib, idarubicin and imatinib were prioritized by PanDrugs. Notably, vandetanib a multi-target protein kinase inhibitor approved for thyroid cancer and currently being tested in clinical trial for AML patients (clinical trial ID: NCT02638428), was prescribed based on the mutations of both *ERBB4* and *FLT4* genes which were already detected in the primary tumour. Based on these results, it could be expected that the administration of any of PanDrugs prescriptions could delay or prevent relapse after standard treatment or in combination.

These results imply that the ITH analysis combined with PanDrugs prescription at early stages could have suggested new therapeutic opportunities to target genes that contributed to the positive selection and relapse after chemotherapy treatment. Additionally, ITH results compared to tumour bulk sequencing results could provide more accurate information for patient treatment that is not being taken into account since variant callers report somatic mutations when VAF is above a certain threshold (typically 10%). Overall, the approach combining temporal ITH analysis plus in silico prescription tools could support evidence-driven clinical decision making providing a personalized drug regimen during the course of the disease.

#### 2.2.2. PanDrugs Prescription in Spatial ITH on TRACERx NSCLC Patients

It has been hypothesized that evaluating the spatial ITH of tumours increases a patient’s therapeutic options [40]. To test this, we performed in silico drug prescription based on mutational profiling obtained from multi-region ITH analysis in NSCLC patients. NSCLC is the most common type of lung cancer accounting for up to 85% of all lung tumours. The standard treatment for early-stage patients (stages I and II) includes surgery resection and adjuvant platinum-based chemotherapy [41]. In addition, advanced NSCLC patients could receive molecular targeted therapies (e.g., EGFRi, ALK inhibitors) according to tumour mutational status. Unfortunately, 33–50% of NSCLC patients develop recurrence [42] so novel therapeutic modalities are urgently required. NSCLC TRACERx consortium has recently investigated the ITH in relation to clinical outcome and clonal evolutionary processes in 100 early-stage NSCLC patients [35]. We applied PanDrugs to the public data corresponding to two early-stage NSCLC TRACERx patients (IDs: CRUK0056 and CRUK0016) in order to identify patients’ therapeutic differences when multi-region sampling and ITH are considered.

**Figure 4:**
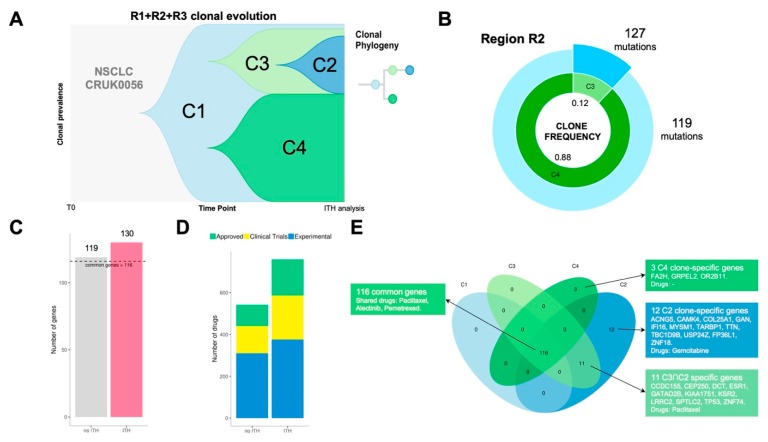
Analysis of the non-small cell lung cancer (NSCLC) patient CRUK0056. Three regions (R1, R2 and R3) were sampled and four different clones (C1, C2, C3 and C4) were detected in this patient. (**A**) Fishplot and phylogeny of the clonal evolution of tumour cell populations. (**B**) Clonal frequencies and number of mutations per clone in region R2. The angles of the doughnut sections represent the frequencies and their radii are proportional to the number of mutated genes per clone. (**C**) Number of altered genes revealed by bulk or intra-tumour heterogeneity (ITH) analysis of R2. We assumed that bulk sequencing would only detect the predominant clone (C4). Bulk analysis exposed 119 mutated genes (25 druggable) while ITH analysis revealed mutations in 130 different genes (31 druggable). The horizontal dashed line indicates that 116 of those genes were mutated in all clones. (**D**) Number of therapeutic candidates revealed by bulk or ITH analysis of R2. We assumed that bulk sequencing would only detect the predominant clone (C4). Drugs have been coloured according to their status. (**E**) Venn diagram of the mutated genes in the tumour clones. PanDrugs proposed paclitaxel, alectinib and pemetrexed to target the clonal mutations (present in all clones). Moreover, other drugs were prescribed to treat the rest of clones based on their specific mutations. C2 was sensible to gemcitabine due to its mutation in *CAMK4*. C2 and C3 shared 11 altered genes including *TP53*, which could have been targeted using paclitaxel. Finally, C4 had mutations in 3 specifics but non-druggable genes.

Patient CRUK0056 was a 71-year-old woman diagnosed with NSCLC (stage IB). She received surgical treatment without adjuvant treatment. Lung tumour tissue from this patient was sampled in three tumour regions (R1, R2, R3). Multi-region whole-exome sequencing showed that tumour clonality composition included four subclone populations (C1, C2, C3 and C4) (Figure 4A).

For every region, we compared ITH versus bulk analysis results (no ITH) which is represented by the most predominant clone detected in each region. Clonal dissection of region R2 uncovered two clonal subpopulations: C3 (12%) and C4 (88%) (Figure 4B). R2 ITH analysis showed a total of 130 point mutations versus 119 detected in the same region when bulk approximation is considered (Figure 4C). PanDrugs study of mutations detected by ITH in R2 exposed 31 druggable genes while R2 bulk analysis revealed 25 druggable targets. Consequently, ITH analysis of R2 expanded the number of candidate drugs to potentially target clones located in R2 region (Figure 4D). In addition, PanDrugs detection of druggable genes in subclones C4 and C3 showed that, although C3 is the least frequent subclone in R2, it harbours more druggable genes (31 genes) than C4 (25 genes). R1 clonal composition include clone C1 (6%) and subclone C4 (94%) while R3 region comprises C1 (2%) and subclone C2 (98%) (Figure S1). In contrast to R2 results, PanDrugs analysis in both R1 and R3 ITH analysis did not increase the number of druggable genes detected. This suggests that the analysis of clone druggability will not involve an expansion in the anti-tumoural drug arsenal in all cases.

Patient CRUK0056 was not a candidate to receive targeted therapies since her tumour was classified as stage IB and she did not harbour any mutation associated to known driver genes (e.g., *EGFR*, *ALK*, *ROS1*, *BRAF*, *RET*). However, the tumour ITH dissection showed a mutational landscape with 116 clonal alterations present in all tumour cells and spatial regions (Figure 4E). Using CRUK0056’s molecular profile, PanDrugs indicated sensitivity to chemotherapies such as paclitaxel, pemetrexed and alectinib (Table S3). All these chemotherapies are approved for the treatment of NSCLC patients and would have worked on the tumour clonal alterations. Additionally, PanDrugs analysis for mutations detected in C2 and C3 also revealed sensitivity to paclitaxel via *TP53* mutation and C2 subclone-specific sensitivity to gemcitabine. In conclusion, all these observations based on molecular evidence suggest that the CRUK0056 patient could have benefited from a treatment based on chemotherapy. These results indicate that the landscape of tumour heterogeneity can be approached therapeutically, and drugs or combinations capable of minimizing the clonal diversity could be identified.

Taken together, these findings show that spatial ITH studies not only allow the detection of minority subclones, they could also be critical to improve in silico prescription performance allowing to discover more potential drug targets while covering as much as possible tumour clonality. Overall, our results indicate that ITH dissection could be a very valuable strategy to increase the therapeutic options in cancer patients.

**Figure 5:**
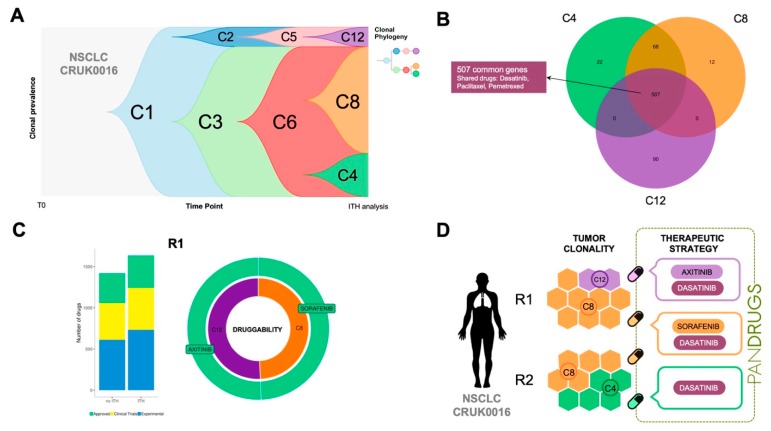
Analysis of the non-small cell lung cancer (NSCLC) patient CRUK0016. Two regions (R1 and R2) were sampled and three different clones (C4, C8 and C12) were detected. (**A**) Fishplot and clonal phylogeny of the evolution of tumoural cell populations. (**B**) Venn diagram of the mutated genes detected in the tumour subclones. PanDrugs proposed dasatinib, paclitaxel and pemetrexed to target the common genes among all clones (trunk). (**C**) Left: Number of therapeutic candidates revealed by bulk and intra-tumour Heterogeneity (ITH) analysis of R1. We assumed that bulk sequencing would only detect the predominant clone (C8). Drugs have been coloured according to their status. Right: Druggability and proposed therapies to treat the clones in R1. Druggability is defined as the percentage of different targetable genes in each subclone over the total targetable genes in a region. (**D**) Therapeutic regimen proposed to treat patient CRUK0016 based on PanDrugs predictions. Dasatinib could be used to target all clones simultaneously (trunk), while axitinib and sorafenib would target clone-specific mutations in C12 and C8 respectively. We did not find any good drug candidate (Drug Score > 0.70) to treat C4 individually. Thus, R1 could be treated with a combination of drugs targeting trunk mutations (dasatinib) and two subclone-specific drugs whereas R2 would be targeted with dasatinib and C8-specific treatment (sorafenib).

To highlight this issue, we propose another example: TRACERx CRUK0016 patient, a 70-year-old man having NSCLC (stage IB) who received adjuvant treatment [35]. CRUK0016 whole-exome sequencing was carried out in two tumour regions (R1 and R2) and ITH dissection revealed three subclonal populations (C4, C8 and C12). Subclones C12 and C8 were present in R1 while C4 and C8 were detected in R2 region (Figure 5A,D). CRUK0016 mutational profiling revealed 507 mutations shared by all subclone populations without known druggable driver genes and a number of clone-specific alterations (Figure 5B). PanDrugs analysis proposed dasatinib targeted therapy (GScore = 0.64, DScore = 0.95) to directly hit *DDR2* (currently in clinical trials for NSCLC) and indicated sensitivity to paclitaxel (GScore = 0.63, DScore = 0.94) and pemetrexed (GScore = 0.79, DScore = 0.84).

In the case of CRUK0016 subclone-specific mutational profiles, PanDrugs expanded the therapeutic arsenal proposing sorafenib and axitinib as the best ranked drugs (DScore > 0.9) to hit subclones C8 and C12, respectively (Figure 5C). Interestingly, sorafenib has shown anti-tumour activity in NSCLC [43] while axitinib is approved to treat kidney tumours and it is currently being tested in clinical trials in NSCLC in combination with additional drugs (i.e., axitinib + avelumab, e.g., clinical trial ID: NCT03472560). A summary of a therapeutic regimen proposal for patient CRUK0016 based on ITH plus PanDrugs results is depicted in Figure 5D. Since we did not find clone-specific drugs for subclone C4 (DScore > 0.7), our proposal includes a combination of drugs to target subclone-specific and clonal alterations that could have been employed to treat this particular patient. In conclusion, PanDrugs results for both CRUK0016 and CRUK0056 patients clearly support the idea that sequential or combinatorial therapeutic regimens will kill a wider spectrum of tumour cells targeting clonal mutations with a drug and subclone populations with additional specific drugs [16].

## 3. Discussion

The cancer precision medicine paradigm is not entirely accomplished yet. Although progress has been made, its successful implementation will rely on effective global strategies for sharing disease-related clinical data standards (which in general are not yet fully defined) as well as on novel methodologies to interpret and integrate patients’ multi-omics profiles with clinical information [44]. Anticancer in silico prescription methods represent a promising group of evidence-guided tools to propose treatments based on tumour genomic profiles, but the systematic performance evaluation of such methods is currently hampered by the lack of public databases with complete clinical data records.

In this study, we present a proof-of-concept reporting the first attempt to systematically evaluate an in silico prescription method using patients’ genomic and clinical data. To do so, we applied PanDrugs on cancer patients’ molecular profiles allowing us to stratify them based on sensitivity/resistant predictions relating them to the survival outcome available in the GDC repository. In particular, patients who received molecular-targeted therapy based on EGFRi were chosen since its prescription is mostly guided by molecular evidence following current clinical guidelines. PanDrugs results in EGFRi-treated patients showed significant Kaplan–Meier plots for OS (*p* = 0.013) exhibiting the consistency amongst PanDrugs predictions and patients’ outcome. Remarkably, PanDrugs was able to a priori identify those potential non-responder patients who showed a worse outcome. Moreover, for these non-responder patients (48.5%) PanDrugs was able to propose alternative therapeutic options. This underlines the usefulness of PanDrugs as a complement tool in clinical decision making.

Nevertheless, our approach is clearly limited in several aspects. In spite of the GDC initiative representing a great effort to make genomic and clinical data accessible and standardised, current clinicopathological annotations are still incomplete. Clinical studies usually incorporate clinicopathological annotation data like age, gender, tumour stage and survival information, however, this is not sufficient. A more complete and curated annotation of patients’ treatment must be systematically collected and improved including full therapeutic regimens, measure of response, drug efficacy, relapse time and overall response. Such annotations are essential to develop computational models for predicting drug response and, consequently, to identify drug response biomarkers and novel drug targets amongst others. Undoubtedly, the performance improvement of anticancer in silico prescription methods critically relies on its systematic evaluation using completely annotated clinicopathological data.

Another current challenge in cancer patient treatment is drug resistance, which is the major reason for therapeutic failure. Drug resistance mechanisms can be either pre-existent (intrinsic) or induced by drugs (acquired) and has been related to ITH [19]. However, drug responsiveness related to ITH is poorly understood and little is known about ITH effects in drug administration and clinical implications [45].

This study applies PanDrugs to mutational profiles obtained by temporal and spatial ITH dissection in AML and NSCLC patients, respectively. Our results indicate that an accurate assessment of ITH might facilitate the development of effective and durable anticancer therapies, but also, to determine patients who will likely benefit from standard therapy and those who will not. Thus, using an AML patient’s genome, we were able to propose approved drugs to hit minority clones (VAF < 0.1%) detected at early stages which were positively selected after chemotherapy causing relapse. Next, subclonal populations detected by multiregion sequencing of NSCLC patients were targeted with different treatments to hit both clonal (trunk) and subclonal alterations suggesting potential drug combinations and personalized therapeutic regimens.

In summary, our findings highlight the need that in silico drug prescription tools should consider: i) the tumour clonal heterogeneity, ii) the allelic frequency of druggable genes, iii) the pre-existence of drug resistance clones and iv) the clonal dynamics of a tumour under selective pressure. We propose here the term “clonotherapy” to describe the optimal therapeutic modalities that would cover individualized intra-tumour heterogeneity. Clonotherapy would cover the concept of targeting clonal alterations (trunk) and subclone populations with different drugs. We hypothesized that clonotherapy will allow the design of precise therapeutic regimens (single-agents, combinations or sequential treatments) anticipating the appearance of relapse, managing drug resistance mechanisms, delaying tumour growth or even induce complete tumour regression.

Nevertheless, we realize that there are a number of limitations related to this work. Firstly, only ~4000 out of >11,000 GDC patients are matched with genomic and clinicopathological data (including drug-response information). When we aim to apply an a priori stratification focused on specific targeted therapy (e.g., EGFRi) the number of patients is drastically reduced to few clinical cases (*n* = 147). This constraint our statistical power, limiting the robustness of our conclusions; for this reason, the present study should be considered as a proof of concept. In addition, we did not perform a comprehensive comparison amongst ITH analysis and standard bulk analysis; instead, we assumed the predominant clone population as an approximation to what would be detected by standard bulk sequencing. Finally, our results suggest that the impact of the clonotherapy approach is variable since in certain cases, the therapeutic options proposed do not differ from bulk standard prescription. This also limits our conclusions and, therefore, a more systematic analysis is needed in future studies.

This study is focused on how in silico drug prescription methods could be improved. To this end, it will be crucial to employ large, standardised and well-annotated patient datasets with genomic and complete clinicopathological data associated. This is mandatory to provide validation datasets for the assessment of novel drug response predictive methods and to identify novel predictive biomarkers based on retrospective studies. In addition, knowing that the current catalogue of known gene–drugs relationships is mostly based on genomic alterations (e.g., single-nucleotide variants (SNVs), CNVs and gene fusions), other information layers must be incorporated [46,47]. For instance, advances in single-cell technologies also represent a promising alternative to bulk sequencing since they offer a comprehensive profiling of tumour genetics, transcriptomics and epigenomics to study clonal architecture and tumour evolution and progression [46,47]. In the present study, we have highlighted the utility of the ITH information layer to propose therapies considering spatial and temporal tumour progression. We believe that this strategy will identify more potential drug targets while covering as much tumour clonality as possible in cancer patients. Since sequencing technologies and bioinformatics methods are evolving and sequencing cost is continuously decreasing, so it is not unreasonable to think that ITH analysis together with clonotherapy will be incorporated into routine clinical practice in the future.

## 4. Material and methods

### 4.1. Datasets and Patients

This study includes 4286 TCGA tumour samples belonging to 4210 patients from GDC data portal [9]. Multi-omic data together with survival and treatment information were retrieved from 32 tumour types. Acute Myeloid Leukemia (LAML) dataset was excluded in this analysis due to the lack of treatment information.

Data from TRACERx lung study [35] was employed to perform the PanDrugs prioritization analysis of candidate therapies based on intra-tumour subclonal heterogeneity. The results reported in this article correspond to the cases CRUK0056 and CRUK0016, two adenocarcinoma patients that were sequenced in 3 (R1, R2 and R3) and 2 (R1 and R2) tumour regions respectively. The subclonal frequencies at each tumour region and information about the mutated genes were extracted from the original article [35]. Mutations located in non-coding regions were filtered out to perform the drug prioritization analysis.

#### 4.1.1. Multi-Omics Data

Mutations, indels, copy-number variants (CNVs) and gene expression data were retrieved from GDC-TCGA patients whose treatments were available. Only those altered genes available in PanDrugs were considered.

Mutations and indels data were obtained from the Xena Browser (https://xenabrowser.net/) [48]. Then, we selected those genomic alterations showing PASS label (MuTect2 annotation) in order to keep those alterations with higher evidence of being somatic. LiftoverVcf from Picard tools 2.0.1 was employed to convert the original GRCh38 coordinates into hg19.

RNA-seq gene expression data z-scores for every gene analysed were collected from cBioPortal web service [49,50]. Only those genes with a z-score > |2| were included in the analysis.

Copy Number Variants segmentation files were downloaded from the Xena Browser. Genes located in the deleted or amplified regions were retrieved using Pyensembl (https://github.com/hammerlab/pyensembl/) for the Ensembl release 93. We only kept those genes with gains or loses supported by expression changes.

#### 4.1.2. Survival Data

Overall survival time (OS) and the corresponding indicator (1-dead, 0-censor) were extracted from the Xena Browser for every patient in the study.

#### 4.1.3. Treatments

Patients’ treatments information was collected from the GDC data portal. Drug nomenclature was standardised using the drug name corrections provided by Gene-Drug Interactions for Survival in Cancer identified (GDISC) [51].

### 4.2. PanDrugs Analysis for NCI-GDC Data

PanDrugs API [7] was employed to prioritize drugs based on TCGA-GDC patient’s mutational and CNVs profiles, taking into account direct targets, biomarkers and pathway member evidence. Drugs administered to the patients were matched to PanDrugs prescriptions. Concordance amongst GDC genomic alterations and PanDrugs annotations were manually verified for EGFRi-treated patients.

### 4.3. Subclonal Drug Prioritization Analysis

Intra-tumour heterogeneity detection analysis was carried out using public whole-genome and whole-exome sequencing data obtained from AML and NSCLC cancer patients [34,35]. PanDrugs methodology was employed to obtain a drug ranking for each subclone type, using the list of mutated genes as input. Then, based on PanDrugs’ DScore and GScore we prioritize those “best therapeutic candidates” that appeared at the top of different rankings and that showed a sensitivity response. When “best therapeutic candidates” were not prescribed, we selected the candidate drugs showing DScore > 0.7 and “sensitivity” response to treat that particular clone. Drug prioritization in bulk versus ITH analysis for NSCLC patients was performed assuming that bulk analysis would only detect the most frequent clone in each tumour region. Therapeutic coverage plots were obtained using *ggplot 2* 3.1.0 and *timescape* 1.6.0 R packages.

### 4.4. Statistical Analysis

Cox’s proportional-hazards models were estimated using the *survival* R package. Kaplan–Meier curves were generated using the *survival* R library for the estimation of the survival function and *survminer* R library for the representation of the curves. Statistical comparison between groups was made using the log-rank test.

## 5. Conclusions

Better tools to predict cancer patient drug response considering inter- and intra-tumour heterogeneity are required. Current in silico drug prescription methodologies are promising tools capable to suggest tailored treatments using patients’ molecular profiles. The accurate evaluation of in silico drug prescription tools require complete and detailed molecular and clinicopathological information. Its improvement needs the integration of additional information layers beyond the tumour bulk mutational profiling such as intra-tumour heterogeneity, that it is widely accepted to be related to poor prognosis and drug response.

Our study is a proof of concept relating in silico drug prescription predictions to patients’ clinical outcomes and also illustrates how to combine intra-tumour heterogeneity information with in silico drug prescriptions to eventually suggest personalized therapeutic regimens.

## Supplementary Materials

The following are available online at https://www.mdpi.com/2072-6694/11/9/1361/s1, Figure S1: ITH analysis of regions R1 and R3 in NSCLC patient CRUK0056, Table S1: List of genes mutated in AML patient UPN933124, Table S2: PanDrugs output for AML patient UPN933124, Table S3: PanDrugs output for NSCLC patient CRUK0056.
