# Supplementary material for: In Silico Drug Prescription for Targeting Cancer Patient Heterogeneity and Prediction of Clinical Outcome
Source: Cancers (Basel). 2019 Sep 13;11(9):1361. doi: 10.3390/cancers11091361 (PMC6769767; doi:10.3390/cancers11091361)
Supplement: Supplementary file 1 [file cancers-11-01361-s001.zip › supplementary_reviewed/cancers-553963 - supplementary-final.docx]

**Supplementary Materials**


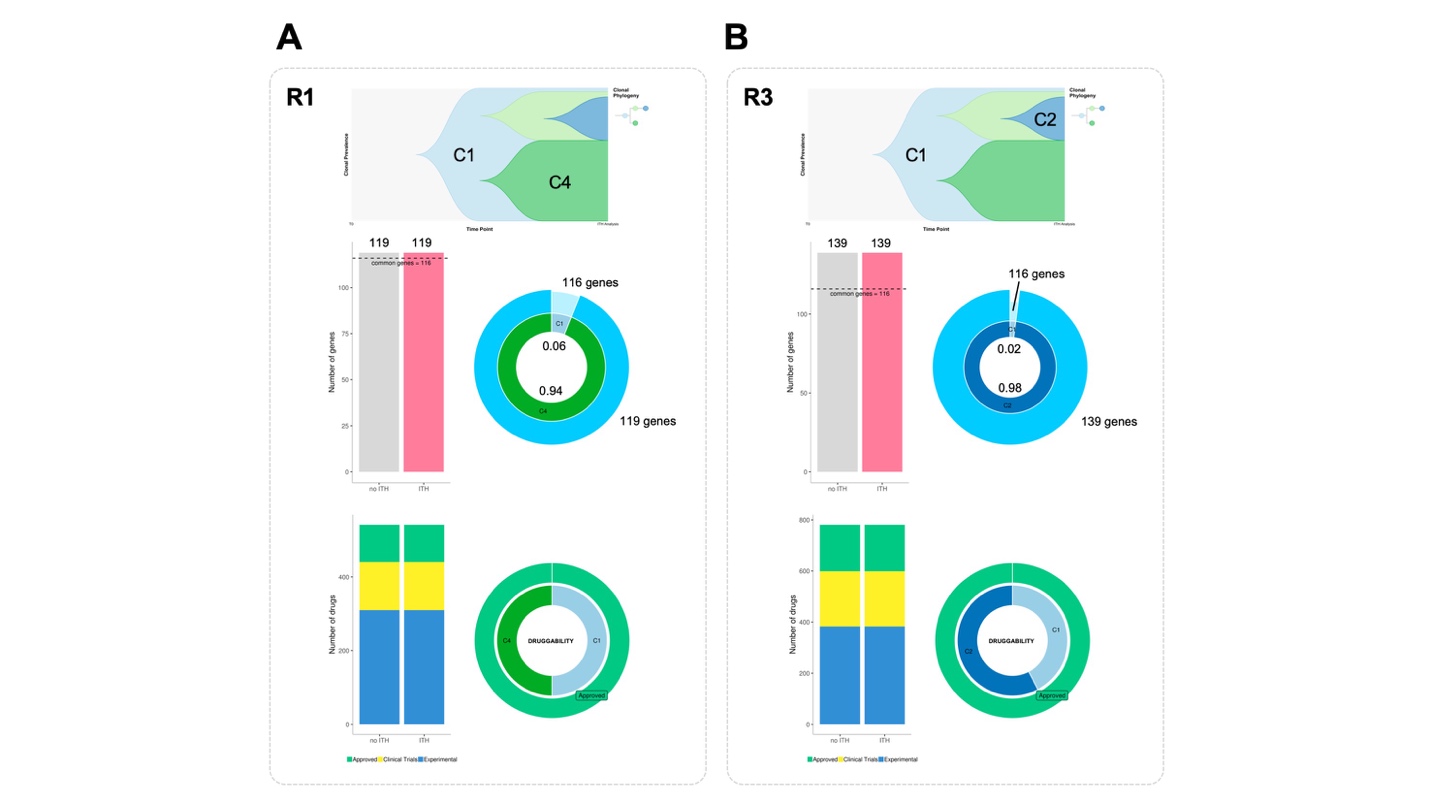


**Figure S1.** ITH analysis of regions R1 and R3 in NSCLC patient CRUK0056. The fishplots and phylogenies (top) represent the clonal evolution of tumour cell populations. The bar plots show the number of altered genes (middle) or the number of therapeutic candidates (bottom) revealed by bulk and ITH analysis. In the later plots, drugs have been coloured according to their status. We assumed that bulk sequencing would only detect the predominant clone in each region. The doughnut plots in the middle represent the clonal frequencies and number of mutations of each region. The angles of the sections and their radii are proportional to the frequencies and the number of mutated genes per clone respectively. The doughnut plots in the bottom show the druggability and proposed therapies to treat the clones. Druggability is defined as the percentage of different targetable genes in each subclone over the total targetable genes in a region. (**A**) R1 contained two clones: C1 (trunk; frequency = 0.06) and C4 (frequency = 0.94) that shared 116 mutated genes. Neither the number of altered genes nor the number of available therapeutic candidates increased after ITH analysis. Although C4 had 3 specific mutations, none of them could be targeted by the drugs in PanDrugs. Thus, both clones had the same druggability. (**B**) R3 contained two clones: C1 (trunk; frequency = 0.02) and C2 (frequency = 0.98) that shared 116 mutated genes. Neither the number of altered genes nor the number of available therapeutic candidates increased after ITH analysis. C2 had 23 specific mutations and 9 of them contributed to increase its druggability with respect to the trunk.
